# Pregnancy associated plasma protein A: An indicator of adverse obstetric outcomes in a South India population

**DOI:** 10.4274/tjod.galenos.2020.05695

**Published:** 2020-04-06

**Authors:** Krupa H Shah, Afsha Anjum, Parvathi Nair, Parvati Bhat, Rajeshwari G Bhat, Shashikala Bhat

**Affiliations:** 1 Melaka Manipal Medical College, Manipal Acedemy of Higher Education, Department of Obstetrics and Gynecology, Manipal, India

**Keywords:** Pregnancy-associated plasma protein a, dual marker test, first trimester aneuploidy screening, adverse pregnancy outcomes, growth restriction of fetus

## Abstract

**Objective:**

First trimester aneuploidy screening (FTAS) has become an integral part of antenatal care in most of centers in India. The serum markers used for FTAS are pregnancy-associated plasma protein A (PAPP-A) and beta human chorionic gonadotropin. In the present study, we aimed to assess the role of PAPP-A in specific adverse fetal maternal events. To analyze pregnancy outcomes with low maternal PAPP-A (≤5^th^ percentile) at the FTAS screening test in southern India, and them compared with a control group of >5^th^ percentile value.

**Materials and Methods:**

A total of 1800 consecutive pregnancies in the first trimester were followed up with PAPP-A levels. The study group consisted 108 subjects, which was compared with a matched control group of 288 subjects. The outcomes considered were spontaneous abortions, fetal anomalies, preterm delivery (PTD), hypertension in pregnancy, intrauterine growth restriction, gestational diabetes, mode of delivery, and birthweight.

**Results:**

For our grouped data, the 5^th^ percentile value for PAPP-A was 0.49 multiple of medians, (incidence-6%). The incidence of fetal major anomalies was higher in the study group [odds ratio (OR): 1.87]. The incidence of minor anomalies, gestational diabetes, and hypertensive disorders was higher in the study group but not statistically significant. The total rate of PTDs (OR:2.1), small-for-gestation-age fetuses (OR:2.3), and low birthweight babies (OR- 2.12) was significantly higher in the study group. We found positive likelihood ratio of 1.4 for PTD, 2 for <5^th^ percentile birthweight, and 1.7 for <10^th^ centile birthweight.

**Conclusion:**

Low PAPP-A pregnancies are at risk of various obstetric complications. Hence, such a pregnancy should have closer surveillance. Further research work on intervention strategy is needed.

**PRECIS:** Using low pregnancy-associated plasma protein A levels in the first trimester of pregnancy, we analyzed the risk of adverse pregnancy outcomes in a South Indian population.

## Introduction

Pregnancy-associated plasma protein-A (PAPP-A) is a glycoprotein that is secreted by the syncytial trophoblast and decidua, and appears in circulation soon after the blastocyst implantation^(1)^. PAPP-A has metalloprotease activity, which cleaves insulin-like growth factor binding protein and releases insulin-like growth factor (IGF). Free IGF, which is ~1% in circulation, has a role in cell multiplication, differentiation, and invasion of trophoblastic cells; hence, it is important for placental development. IGF also regulates the uptake of glucose and amino acid, thus fetal growth^(2,3)^. Accordingly, PAPP-A is a part of the system that controls IGF activity^(4)^. There is growing evidence that low PAPP-A levels in the first trimester are associated with adverse fetal and maternal outcomes such as spontaneous abortion (SA) preterm delivery (PTD), fetal growth restriction (FGR), preeclampsia (PE), and stillbirth, apart from chromosomal aneuploidy^(5,6,7)^.

Low PAPP-A is defined as a maternal serum concentration less than the 5^th^ percentile. The landmark randomized clinical trial FASTER found that the PAPP-A of <5^th^ percentile was associated with FGR [odds ratio (OR)-3.2), SA (OR-2.5), PTD (OR-1.9], birthweight below the 5^th^ percentile (OR-2.8), PE (OR-1.5), and placental abruption (OR-1.8)^(8)^. Other cohort studies have confirmed similar findings^(9,10)^.

The aim of the present study was to obtain the 5^th^ percentile value in our grouped data, and to analyze the risk of adverse pregnancy outcomes in ≤5^th^ percentile in the PAPP-A group in reference to the >5^th^ percentile group.

## Materials and Methods

The study was approved by the Manipal Ethics Committee, Manipal Academy of Higher Education (approval number: MUEC/03/2015). This prospective study was conducted at Dr. TMA Pai Hospital, Udupi, affiliated to Manipal University, Manipal from November 2015 to 2018. Informed consent was obtained after briefing the participants about the study in Kannada and English languages. Gestational age was based on the last menstrual period with previous regular cycles and corresponding scans between 7-10 weeks of gestation or on measured crown-rump length (CRL) at 7-10 weeks of gestation. A disparity in CRL of up to 5 days was considered acceptable. We excluded multiple pregnancies, patients with renal diseases, chronic hypertension, insulin-dependent diabetes mellitus, cardiac disorders, and chromosomal abnormalities. All singleton pregnancies undergoing first trimester aneuploidy screening were enrolled in the study. Information regarding age; weight; height; first trimester events such as threatened abortion, and previous history of fetal aneuploidy were obtained. The smoking status was also determined, even though it was uncommon.

Three to four milliliters of venous blood sample was collected. The serum was separated and analyzed at the biochemistry department of Kasturba Hospital, Manipal. The test was conducted using an automatic analyzer with an electro-chemiluminescence assay on a Cobase 601 analyzer, which provided marker concentration. Risk calculation was performed using the SSWD Lab version 5.0 software, and values are given as multiple of medians (MoM).

The percentile values for PAPP-A were obtained from 1800 enrolled pregnancies for FTAS. All women with serum PAPP-A of less than the 5^th^ percentile were enrolled as cases, and women with normal (>5^th^ percentiles) PAPP-A were taken as controls. The fetal aneuploidy risk of 1:250 or higher was taken as high risk for chromosomal abnormality and hence, invasive test counseling was offered. All the confirmed chromosomal abnormalities were counseled regarding the outcome of the fetus and excluded from the study. The rest of the women were followed as per the hospital protocols. Woman detected with lethal anomalies at the second trimester scan were given the option for termination of pregnancy (TOP). Pregnancy outcome information was obtained for SA, fetal anomalies, PTD, hypertension in pregnancy (PIH), PE, gestational diabetes (GDM), small-for-gestational-age (SGA) babies, delivery mode, and low birth weight babies (LBW).

SA was defined as loss of pregnancy before 20 weeks of gestation or a fetus weighing <500 g. PTD was defined as a delivery before 37 completed weeks. SGA was defined as birth weight less than -2 SD below the gestational age. The reference for percentile birth weight was taken from Callen’s book of ultrasound.^(11)^ PIH was determined as a blood pressure of more than 140/90 mm Hg after 20 weeks of pregnancy in two recordings 6 hours apart, and without significant proteinuria. PE was determined as hypertension with proteinuria >300 mg/24 hours or any sign of end-organ damage^(12)^. LBW was defined as a birth weight of less than 2.5 kg^(13)^.

### Statistical Analysis

Statistical analysis was performed using The Statistical Package for the Social Sciences (SPSS) software version 20, and p values <.05 were considered statistically significant after application of the chi-square test. Student’s t-test was used for the comparison of means. Fisher’s exact test was used when appropriate. OR with 95% confidence intervals was calculated for several outcomes. The performance characteristics (sensitivity, specificity negative and positive predictive values, likelihood ratio) were calculated. Receiver operating characteristics curves (ROC) were constructed and the area under the curve was calculated for certain pregnancy complications.

## Results

**Figure 1:**
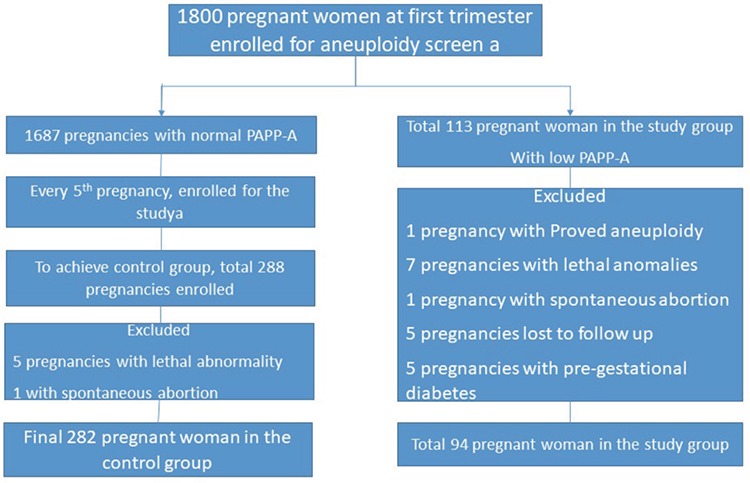
Consort diagram about enrolling subjects and sample size in the different group

A total of 1800 pregnant women attending the antenatal clinics underwent first trimester fetal aneuploidy screening and the 1^st^, 3^rd^, 5^th^, 10^th^, 50^th^, 90^th^, 95^th^, and 99^th^ percentile values for PAPP-A obtained in present cohort were 0.23, 0.33, 0.49, 0.61, 1.26, 2.4, 3.13, and 5.1 MoM. The 5^th^ percentile of PAPP-A values corresponded to 0.49 MoM in the study population. The incidence of low PAPP-A was 6%. A total of 113 pregnancies with a PAPP-A value ≤0.49 constituted the study group. Amongst them, one had aneuploidy fetus, one had a SA at the 15^th^ week of gestation, seven had a TOP in view of lethal anomaly, five had pre-gestational diabetes, and five were lost to follow-up, which were excluded for delivery outcome analysis. Accordingly, 94 patients were followed until delivery and then analyzed. Two hundred eighty-two (1:3) pregnant women were enrolled in the control group with normal PAPP-A values; every 5^th^ pregnancy from a cohort of 1687 subjects was taken to form the control group. The consort flow diagram (Figure 1) depicts the subject enrolment. We observed five fetuses with cardiac anomalies (transposition of the great vessels n=1, atrio-ventricular wall defect (n=2, double-outlet right ventricle (n=1, hypoplastic left heart (n=1), one fetus with a urogenital abnormality, and one pregnancy with severe oligohydramnios at 18 weeks of gestation in the study group. We had a total of six pregnancies with an increased risk of fetal aneuploidy and prenatal diagnosis revealed one fetus had Patau syndrome. There were five lethal anomalies in the control group, three involving the central nervous system (agenesis of the corpus callosum, severe hydrocephalus, vermian agenesis), one cardiac anomaly (double-outlet right ventricle), and one with a renal anomaly. Accordingly, 288 patients were enrolled in the control group to obtain 282 controls for analysis, because there was one second trimester abortion.

The characteristics of both groups are presented in Table 1. Parameters such as age, weight, weight gain in pregnancy, and sampling period were comparable in both groups. The mean body mass index (BMI) was 22.9±3.91 kg/m^2^ for the study group, whereas it was 21.7±3.87 kg/m^2^ in the control group, which was found to be significantly higher. There were 170 (60%) nulliparous patients in the control group and 45 (49%) in the study group, the difference was not statistically significant. The mean CRL was 58.36±5.5 mm in the study group and 60.0±6.4 mm in the control group (p=0.051). Table 2 compares the obstetrics complications with ORs for various pregnancy complications. The incidence of threatened abortion (10.6% vs. 11.3%) and premature rupture of membrane (7.4% vs. 8.5%) was not significantly different between the study and control groups. We found a higher incidence of minor birth defects (6.4% vs. 4.3%) in the study group, which was not statistically significant. Table 3 mentions the association of low PAPP-A with pregnancy outcomes. Table 4a and 4b depicts the predictive value of PAPP-A for various complications. ROC analysis suggested further predictive ability.

## Discussion

We took percentile values for the study, another way is to fix arbitrary absolute values for the formation of the study group and analysis. In our study, the cut-off value was 0.49 MoM, which is in concordance with study by Patil et al.^(14)^ Low PAPP-A is considered between 0.3-0.5 MoM in different studies.^(15,16,17)^ The incidence of low PAPP-A (<5^th^ percentile) was 6% in our study, the study by Yaron et al.^(16)^, reported 15.4%, such a variation may be due to different populations and patient profiles. The incidence was 5.4% in a study by Cooper et al.^(18)^, with an absolute cut-off for PAPP-A <0.4 MOM.

We observed a higher incidence of SA (1.1% vs. 0.7%) in the study group, which was not statistically significant. The study by Barrett et al.^(15)^ observed increased pregnancy loss and Kaijomaa et al.^(19)^ reported an OR of 7.7 for SA in the low PAPP-A group, suggesting a strong risk factor. An abortion has diverse etiology and one important reason is depleted hormonal and nutritional support to a growing fetus. Here, the low amount of PAPP-A could be one of the reasons for suboptimal placental growth and function leading to the above-mentioned insufficiency.^(10,17)^ Another reason could be undetected chromosomal abnormalities becoming naturally aborted in this case.

We observed a higher number of congenital lethal abnormalities in targeted scans in the study group (OR: 3.7); such increased chromosomal/non-chromosomal birth defects were observed by Barrett et al.^(15)^ (RR: 2.2), even after excluding pregnancies with chromosomal aneuploidy. All 12 patients with major birth defects underwent TOP and fetal autopsy; fetal chromosomal status was known for only a few fetuses. There was no statistically significant difference between the groups for minor anomalies, a similar observation was documented by Barrett et al.^(15)^ We observed cardiac defects as a major birth defect in the study group, and mainly nervous system abnormalities in the control group. Such an observation is not present in the available literature, hence, this could be coincidence. However, further studies are required to know the type of fetal malformations in women with low PAPP-A.

After excluding all birth defects and chromosomal abnormalities, a significant increase risk of PTD, SGA and LBW was found in the study group. The maximum risk increase was about 3 folds for SGA (OR: 2.88). These findings dovetail with other studies^(8,10,15)^. However, there is a report mentioning its non-association with both PTD and IUGR^(20)^. The study by Pummara et al.^(3)^ suggested that <10^th^ PAPP-A percentile was associated with idiopathic preterm and concluded that such pregnancies should be labelled as high risk for preterm delivery. A systemic review and meta-analysis by Morris et al.^(10)^ also confirmed a moderate association of low PAPP-A with SGA and PTD with poor the predictive ability. We found a positive likelihood ratio (LR) of 1.4 for PTD, 2 for <5^th^ percentile birth weight, and 1.7 for <10^th^ percentile birthweight. A similar positive LR of 1.84 for PTD, 2.65 for <5^th^ percentile birth weight, and 1.96 for <10^th^ percentile birth weight was revealed by Morris et al.^(10)^ Our findings are suggestive of low sensitivity but high negative predictive value for the prediction of adverse events, which are in concordance with the FASTER trial^(8)^.We had one stillbirth of 900 g at 30 weeks of gestation in the study group, which was not statistically significant. The incidence of PIH was higher in the study group (14.9% vs. 9.2%); however, it was not statistically significant. A similar positive but strong correlation was found in various studies^(7,17)^. PIH can be explained by defective placentation and inflammatory cascade activation resulting in different complications. The study by Beneventi et al.^(21)^ suggested that first trimester PAPP-A levels were significantly lower among the pregnancies subsequently affected by gestational diabetes. We also noticed a higher incidence of GDM (16% vs. 9.2%), which was not statistically significant. A study by Petry et al.^(4)^ suggested the possibility of a link between low PAPP-A concentrations and glucose levels due to the effect of IGF on insulin sensitivity.

We believe that the root cause for the increased incidence of PTD is placental insufficiency, IUGR/SGA, and LBW. The placental syncytio-trophoblasts produces of PAPP-A, which acts as one of the proteins for the prevention of the recognition of the fetus by the maternal immune system, helps in angiogenesis, placental cellular hyperplasia, and maternal vascular system invasion by trophoblasts. It also helps in matrix mineralization; therefore, low PAPP-A is linked to the rejection of fetus and unhealthy placentation resulting in a spectrum of complications. Low PAPP-A has been accepted as a marker for pregnancy complications, and it is recommended to follow such patients for growth disorders^(22)^.

Limitations and strengths: this study provides useful information to physicians that can help them in the management of high-risk pregnancy with normal karyotype and low PAPP-A. The PAPP-A values cannot be extrapolated because it represents the Southern region of India. The higher BMI in the study group can be a confounding factor. Due to the small sample size, only few events could be registered for certain pregnancy outcomes.

## Conclusion

PAPP-A levels can be used to differentiate pregnancy as high risk for adverse obstetric outcomes. Low PAPP-A has a modest association with adverse pregnancy outcomes in the absence of chromosomal abnormalities. The awareness of low values can help in improving fetal outcomes by closer surveillance for adverse events. However, it has limited use due to its lower predictive value. Further research on the development of a prediction model and formation of a preventive strategy for different obstetric adverse events is needed.

## Figures and Tables

**Table 1:**
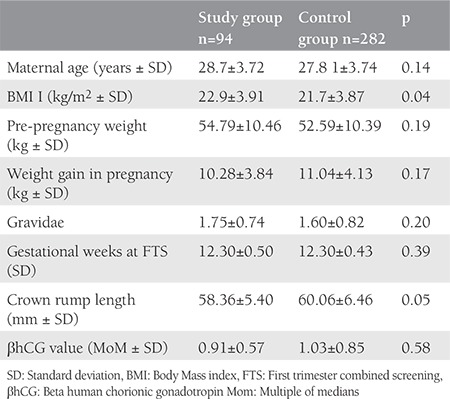
Characteristics of study population in subgroups

**Table 2:**
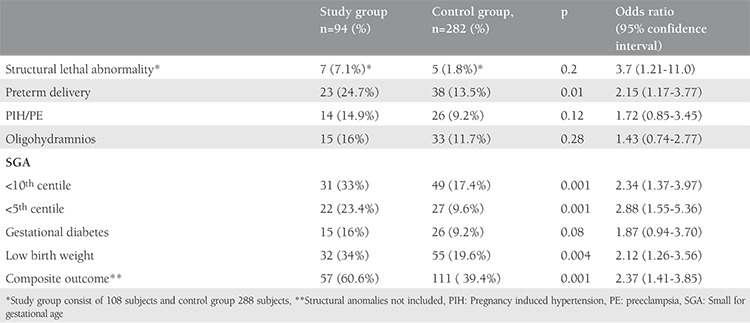
Complications during pregnancy in both the groups and predictive ability of marker

**Table 3:**
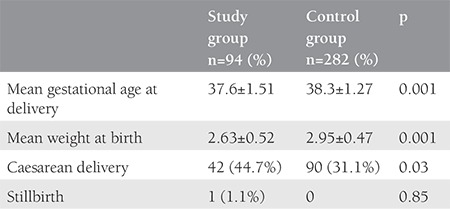
Outcome of pregnancy in both groups

**Table 4a:**
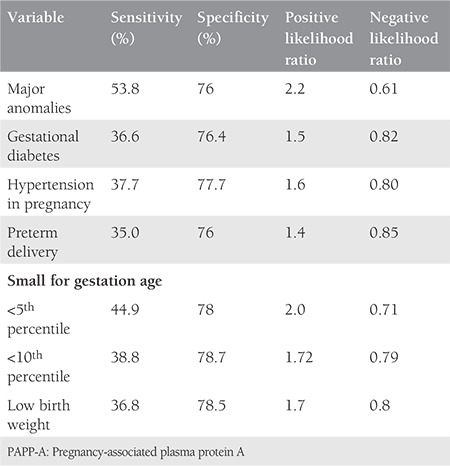
Performance characteristics of low PAPP-A for selected outcomes

**Table 4b:**
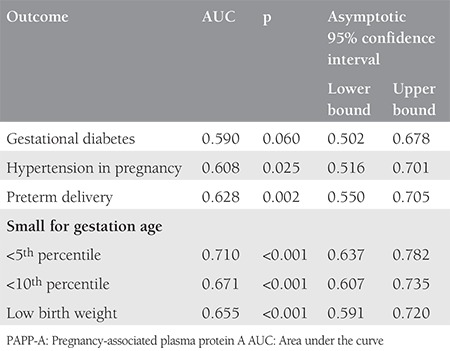
Performance characteristics of low PAPP-A for selected outcomes
